# Customized protective palatal obturator for intubation in newborns in cleft lip surgery: a randomized controlled trial

**DOI:** 10.1080/07853890.2025.2561802

**Published:** 2025-09-22

**Authors:** Michaela Richtrová, Olga Košková, Petr Marcián, Libor Borák, Tereza Bönischová, Dominik Fabián, Martin Janků, Marek Joukal, Kateřina Vymazalová, Petr Štourač

**Affiliations:** ^a^ Department of Paediatric Anaesthesiology and Intensive Care Medicine, University Hospital Brno, Brno, Czech Republic; ^b^ Faculty of Medicine, Masaryk University, Brno, Czech Republic; ^c^ Department of Burns and Plastic Surgery, University Hospital Brno, Brno, Czech Republic; ^d^ Faculty of Mechanical Engineering, Institute of Solid Mechanics, Mechatronics and Biomechanics, Brno University of Technology, Brno, Czech Republic; ^e^ Department of Simulation Medicine of the Faculty of Medicine, Masaryk University, Brno, Czech Republic; ^f^ Faculty of Medicine, Department of Anatomy, Masaryk University, Brno, Czech Republic

**Keywords:** Cleft lip, newborns, protective obturator, 3D printing, intubation, tissue damage, anesthesia management, patient specific approach

## Abstract

**Background:**

Orofacial clefts are common congenital malformations, affecting both facial aesthetics and function. Intubation in newborns with cleft lip and palate is challenging and carries a high risk of oral tissue damage. This study investigates the use of a customized protective palatal obturator (CPPO) to improve intubation safety and reduce tissue injury during cleft lip surgery.

**Methods:**

A single-center, randomized neonatal sub-study was conducted, including 55 newborns who underwent cleft lip surgery. Patients were randomized into an intervention group (CPPO use) and a control group (standard intubation without CPPO). The primary aim was to evaluate the degree of oral tissue injury during intubation, its severity, and location, in both groups, secondary aims included laryngoscopy image during intubation (modified Cormack-Lehane scoring system), intubation time, and attempts, number of intubations attempts and anesthesiologic complication during intubation. This study was registered on www.clinicaltrials.gov (ClinicalTrials.gov Identifier: NCT04422847 and NCT04422964).

**Results:**

No tissue damage occurred in the CPPO group, while the control group had a 21.4% incidence of tissue injury (*p* = .023). Secondary outcomes showed no statistically significant differences between groups for intubation time or the number of intubation attempts. Difficult intubation was less frequent in the CPPO group (40.7%) compared to the control group (50%), though this difference was not statistically significant.

**Conclusion:**

The CPPO significantly reduces the risk of tissue damage during intubation in newborns undergoing cleft lip surgery, without increasing intubation time or attempts. It is particularly beneficial for severe clefts, and its use may facilitate safer airway management in these high-risk patients.

## KEY MESSAGES

- Customized protective palatal obturators enhance intubation safety in newborns with cleft lip, alveolus and palate.
- The customized protective palatal obturator is especially advantageous for patients with more severe clefts, offering better protection against tissue damage during intubation.
- The customized protective palatal obturator does not prolong intubation time or increase the number of intubation attempts, making it an effective tool for better airway management without added difficulty or delay.

## Introduction

Orofacial clefts are among the most common congenital malformations. The average incidence of orofacial clefts is now 1 in 700 live births [1]. Orofacial clefts affect not only the aesthetics of the face, but especially the function; therefore, a multidisciplinary approach and centralization of care into cleft centers are necessary for successful treatment. Cleft centers worldwide have their own treatment protocols and timing for primary surgeries [2]. The integration of advanced technologies such as 3D printing offers promising solutions for personalized treatment approaches given the complexity of orofacial cleft management, especially during surgical interventions [3]. In the Cleft Center of the University Hospital Brno (UHB) in the Czech Republic, about 80 newborns with orofacial cleft are taken into care per year. Typically, cleft lip surgery is performed from the 2nd day of life and cleft palate surgery is recommended from the 7th month of patient’s age [4].

Newborns with malformations such as cleft lip and palate are a special group of patients who can be expected to have difficult airway management [5]; therefore, anesthesia care for newborns is usually performed in specialized children’s anesthesiology centers. Pediatric patients with orofacial clefts are considered at high risk for difficult airway management due to facial malformations and changes in the upper airway. In addition, among pediatric patients with orofacial clefts there are also children with hereditary syndromes, which also increases the risk of difficult airway management.

The concept of using protective devices during intubation for cleft patients is not entirely new. Previous approaches have included the use of pre-formed hard shields and custom-made flexible materials to cover the defect [6,7]. However, the application of a high-precision, 3D-printed, patient-specific approach in the distinct neonatal cleft lip and palate population has not been previously investigated. Based on our observations, tissue damage of the lip, alveolar segments or palate frequently occurs because of laryngoscope blade manipulation in the oral cavity during intubation, which is undesirable for further healing and recovery after surgery [8,9]. To increase the comfort of the anesthesiologist during neonatal intubation and to reduce tissue damage, a customized protective palatal obturator (CPPO) can be used. The CPPO were initially developed using cadaver models (see Appendix for the details on the early development of the obturator), but they have since become fully functional tools utilized in live newborns. By exploring these factors, the study aims to provide a comprehensive evaluation of the CPPO’s effectiveness in improving intubation safety and outcomes for newborns with cleft lip, alveolus and palate (CLAP).

The primary aim of the study was to assess how CPPO reduces the risk of oral cavity tissue damage during intubation. In addition to the primary objectives, we also examined several secondary outcomes. Specifically, we investigated whether CPPO is more effective for intubating more severe types of clefts compared to less severe clefts, does not prolong intubation duration, does not necessitate more intubation attempts, and does not impact the laryngeal view during video laryngoscopy.

## Materials and methods

### Study design and participants

This prospective, single-center, single-blind randomized study was approved by the Ethics Committee of the University Hospital Brno (Approval Number: 05-100620/EK, date of approval 10.6.2020), and it was registered on www.clinicaltrials.gov (ClinicalTrials.gov Identifier: NCT04422847 and NCT04422964). We performed a subgroup analysis of patients who underwent cleft lip surgery specifically during the neonatal period and met the inclusion criteria of both studies registered on ClinicalTrials.gov. Written informed consent and consent for publication was obtained from all parents or legal guardians of the study participants. We conducted this trial following the principles of Good Clinical Practice and the Declaration of Helsinki. No changes to the study protocol occurred after trial commencement. The manuscript was presented following the Consolidated Standards of Reporting Trials (CONSORT) 2010 statement [10].

**Figure 1.**
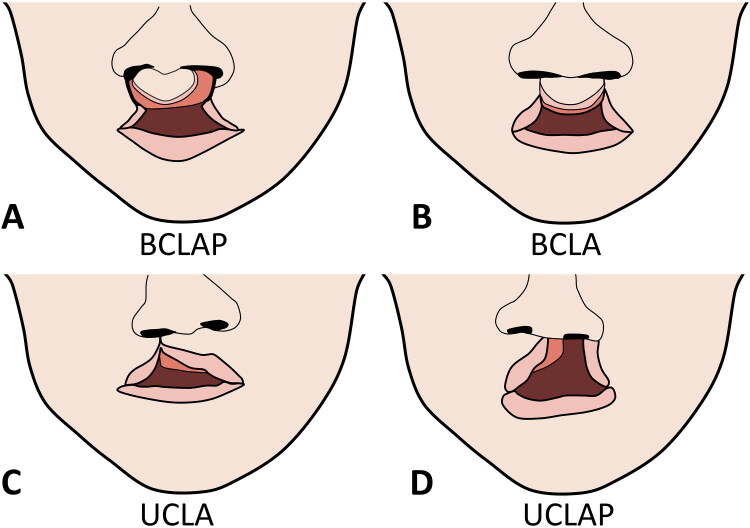
Cleft types: (A) BCLAP - bilateral cleft lip, alveolus and palate. (B) BCLA - bilateral cleft lip and alveolus. (C) UCLA - unilateral cleft lip and alveolus. (D) UCLAP - unilateral cleft lip, alveolus and palate.

All patients who underwent primary cleft lip surgery between June 2020 to November 2023 in the Cleft Center of The University Hospital Brno were considered eligible for inclusion. Inclusion criteria for this study were set as follows: pediatric patients with unilateral or bilateral cleft lip and alveolus (U/BCLA) or unilateral or bilateral cleft lip, alveolus and palate (total cleft; U/BCLAP) who underwent primary cleft lip reconstruction within 0–28 days of age (Figure 1; details are provided also in Zenodo repository, doi:10.5281/zenodo.15014681). Patients were excluded from the study because of the following conditions: patients with only unilateral cleft lip without cleft alveolus (UCL) or bilateral cleft lip without cleft alveolus (BCL), patients with genetically confirmed syndrome disability with a risk of complicated airway management due to anatomical abnormalities despite of cleft defect itself, patients with atypical clefts of the face, patients with cleft lip and alveolus (CLA) and cleft lip and palate (CLAP) who underwent primary cleft lip reconstruction later than at 28 days of age, patients with airways secured preoperatively (tracheostomy), patients on artificial lung ventilation, patients with coagulopathy, thrombocytopenia/thrombocytopathy, patients at risk of malignant hyperthermia, patients for whom the consent of legal representatives to the research project has not been obtained.

Patients, who met inclusive criteria, were randomized with a 1:1 allocation by an independent administrative worker, was not involved in active treatment of the patients, to the intervention group (with CPPO) and the control group (standard procedure without CPPO). Description of both groups is provided in Table 1.

**Table 1.** Baseline data.

Group	Type of clefts	Incidence	Gestation week	Birth weight (g)	Age at the time of surgery (days)	Sex (number of M/F)
Intervention	UCLA	9 (33.3%)	40 (37 − 42)	3720 (2800 − 4340)	9 (5 − 25)	5/4
	BCLA	0 (0%)	n/a	n/a	n/a	n/a
	UCLAP	11 (40.7%)	40 (37 − 42)	3690 (2300 − 4230)	11 (5 − 26)	7/4
	BCLAP	7 (25.9%)	40 (39 − 41)	3400 (2764 − 4320)	11 (6 − 15)	5/2
Control	UCLA	9 (32.1%)	40 (34 − 41)	3300 (2100 − 4000)	8 (5 − 25)	3/6
	BCLA	1 (3.6%)	39 (39 − 39)	2842 (2842 − 2842)	6 (6 − 6)	1/0
	UCLAP	12 (42.9%)	39.5 (37 − 40)	3375 (2550 − 3590)	8.5 (3 − 24)	7/5
	BCLAP	6 (21.4%)	39.5 (37 − 41)	3135 (2810 − 3600)	11.5 (9 − 17)	5/1

Cleft incidence and median (min-max) values of gestation week, birth weight and age at the time of surgery for both intervention and control groups.

### CPPO design and 3D printing

**Figure 2.**
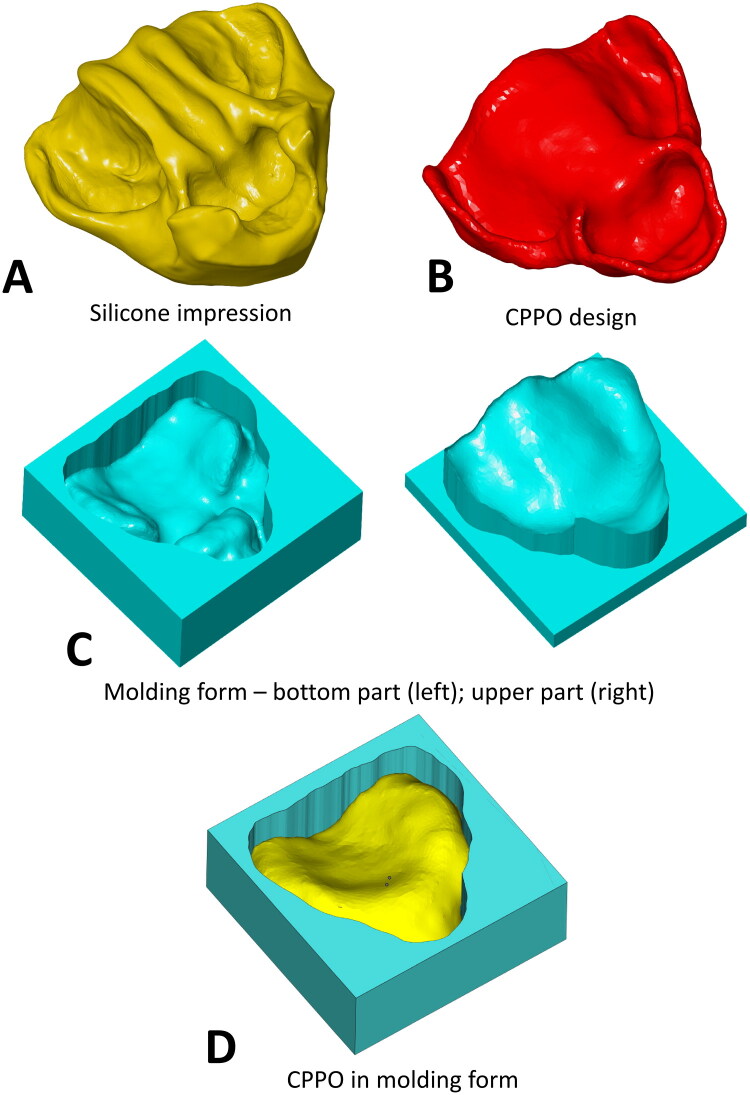
Design workflow of the customized protective palatal obturator (CPPO). (A) the initial silicone impression taken from the patient’s upper jaw, capturing the specific anatomy of the cleft. (B) The final digital 3D model of the CPPO, created using CAD software. (C) CAD design of two-part mold. (D) The final CPPO being cast from biocompatible material within the assembled mold prior to removal.

For the design of the CPPO model, real silicone impressions were used for all patients, capturing the details of the maxillary cleft (defect and alveolar arch) [11]. The silicone impression was then scanned using a 3D scanner (Shining3D EinScan SE, SHINING 3D Technology GmbH., Stuttgart, Germany) whose accuracy of 0.1 mm was sufficient for CPPO design purposes. Using Computer Aided Design (CAD) software Meshmixer (Meshmixer, Autodesk, San Rafael, CA, USA) and Materialise Magic 22.0 (Materialise, Lueven, Belgium), the geometry was adjusted so that the CPPO not only fit the patient′s palate but also fit sufficiently behind the alveolar ridge towards the patient’s scar. This prevents possible slippage of the CPPO during intubation. Furthermore, it is not necessary to replicate the detailed geometry of the defect. It is sufficient to simply patch it and remove the remaining goitre after impression. Subsequently, a mold was created using the CPPO geometry and printed using a 3D printer (FDA 3D printer Prusa I3 MK3S). For molding purposes, a certified material for use in the oral cavity was used (Figure 2). CT/MRI or Cone-Beam Computed Tomography (CBCT) imaging data could also be used, but their accuracy is insufficient for CPPO design purposes [12,13], and examinations are only performed in exceptional cases.

### Evaluation process

**Figure 3.**
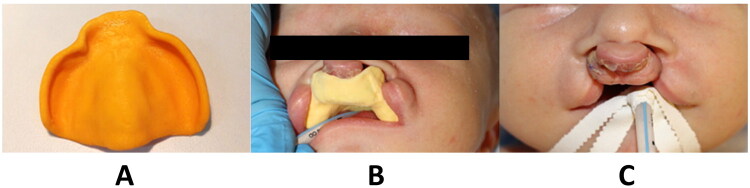
Clinical demonstration of the CPPO used during the intubation process of a newborn with BCLAP. (A) CPPO. (B) a patient with the CPPO correctly seated on the upper jaw, demonstrating coverage of the underlying palate, alveolar ridge, and premaxilla. (C) Frontal view of an intubated newborn prior to cleft lip surgery.

All patients included to the study were intubated with video laryngoscope (KARL STORZ C-MAC^®^ 8403ZX monitor and D-BLADE MAC^®^ pediatric) and the anesthetic management was standardized in all cases. In the intervention group, the CPPO was inserted into the oral cavity under deep inhalation anesthesia during spontaneous ventilation, immediately prior to tracheal intubation (Figure 3). In the control group, no obturator was used. The primary outcome of the study was the assessment of oral tissue damage during intubation, its severity, and location. In all cases, the severity of oral cavity tissue damage was evaluated using a modified classification according to Mourão [8] into 3 degrees—degree 1: presence of swelling or petechiae (without damaging continuity of upper skin or mucosa layer), degree 2: bleeding (with damaging continuity of upper skin or mucosa layer) of soft tissue in the entire cleft defect area, degree 3: premaxilla fracture in BCLA and BCLAP patients. Tissue damage was also categorized based on its localization into three groups: 1: Lip, 2: Alveolus, and 3: Palate/Vormer area, with a single damage possibly being classified into multiple groups.

Secondary outcomes of the study included intubation conditions, including the laryngoscopic view during intubation, intubation time, and the number of intubation attempts. Therefore, the time to achieve successful intubation and the number of intubation attempts were recorded. Successful intubation was defined as the first wave of end-tidal CO_2_. Moreover, the difficulty of the intubation process was predicted before the intubation itself using the modified Cormack-Lehane scoring system (MCLS) [14]. This score describes the view obtained at direct laryngoscopy as follows: 1 = Full view of the glottis, 2a = Partial view of the glottis, 2b = Arytenoids or posterior part of the vocal cords only just visible, 3 = Only epiglottis visible, 4 = Neither glottis nor epiglottis visible. MCLS types 2b, 3, and 4 represent difficult intubation [14]. To minimize potential inter- and intra-observer variability associated with this scoring system, all assessments were performed by a dedicated team of anesthesiologists experienced in intubation of patients with cleft lip and palate.

### Statistical analysis

Primarily, we monitored the correlation between the use of the CPPO and tissue damage. Furthermore, we examined the relationships between the use of the CPPO and intubation times, the number of intubation attempts and MCLS scored during intubation. The collected data were then statistically analyzed, assessing the null hypotheses that the distributions/central tendency or frequencies between groups do not differ significantly (i.e. that are the same). For this purpose, the Mann-Whitney U test was employed for numerical variables, while the Pearson chi-square test or Fisher’s exact test was utilized for categorical variables. The selection of the specific test depended on the expected frequencies in the contingency table. Fisher’s exact test (FET) was preferred when more than 20% of the cells in the contingency table had expected frequencies less than 5, or when any cell had an expected frequency less than 1. A significance level of α = 0.05 was applied across all tests. Consequently, a p-value < 0.05 resulted in the rejection of the null hypothesis, indicating a statistically significant difference between the groups. The Shapiro-Wilk test indicated that the numerical variables were not normally distributed (*p* < .05). Statistical analyses were conducted in Statistica 13.5 (STATISTICA software TIBCO Software, Tulsa, OK, USA).

## Results

**Figure 4.**
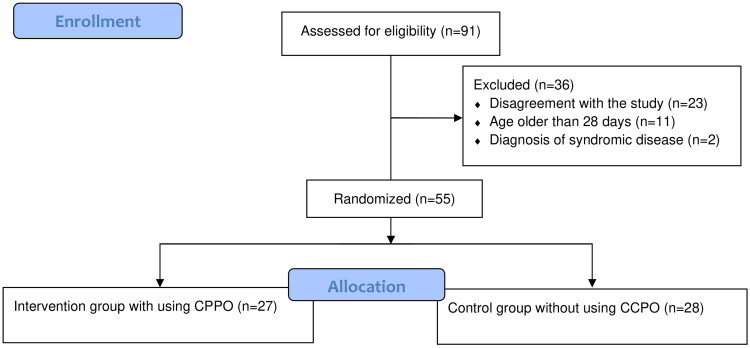
Flowchart illustrating patient enrollment, exclusions, and final allocation to intervention and control groups.

Out of 91 patients that underwent primary cleft lip surgery, only 55 patients met inclusive criteria and were randomized to the intervention group with CPPO (*n* = 27) and the control group without CPPO (*n* = 28). Figure 4 shows a flowchart of the process of patient enrollment. The median age at the time of surgery was 10 days (min-max: 3–26). The median gestational week at birth was 40 (min-max: 34–42). The median body weight was 3380 grams (min-max: 2100–4340). The type of cleft was determined for all patients and a detailed description of the both groups is provided in Table 1. U/BCLA was considered as a less severe cleft type, U/BCLAP as a more severe cleft type.

The primary outcome of this study was the incidence, severity, and localization of tissue damage during intubation, as detailed in Table 2. This table presents the number and percentage of patients experiencing damage at various locations (lip, alveolus, palate, vomer) and the degree of damage (graded 0–3) in both the intervention (CPPO) and control groups. Secondary outcomes included the MCLS score, reflecting the laryngoscopic view during intubation (Table 3), and the number of intubation attempts required (Table 4). These tables provide a comparison of these parameters between the intervention and control groups. The results of the statistical analysis of the evaluation of the primary and secondary objectives are summarized in Table 5.

**Table 2.** Localization and degree of tissue damage.

	The intervention group—with CPPO *n* = 27	The control group—without CPPO *n* = 28	*p*-value*
Tissue damage (degree)			
0	27 (100%)	22 (78.6%)	.023**
1	0 (0%)	3 (10.7%)	.236
2	0 (0%)	1 (3.6%)	1.000
3	0 (0%)	2 (7.1%)	.491
Localization (multiple locations possible)			
lip	0	3	n/a
Alveolus	0	4	
Palate	0	1	
Vomer	0	0	

Notes.

* *p*-values are calculated using Fisher’s Exact Test.

** *p* < .05 suggests a significant association between tissue damage and CPPO use.

**Table 3.** Modified Cormack-Lehane scores.

MCLS	The intervention group—with CPPO *n* = 27	The control group—without CPPO *n* = 28	*p*-value*
1	4 (14.8%)	5 (17.9%)	1.000
2a	12 (44.4%)	9 (32.1%)	.412
2b	9 (33.3%)	11 (39.3%)	.781
3	2 (7.4%)	3 (10.7%)	1.000
4	0 (0%)	0 (0%)	n/a

Notes.

* *p*-values are calculated using Fisher’s Exact Test.

**Table 4.** The number of intubation attempts.

Number of intubation attempts	The intervention group—with CPPO *n* = 27	The control group—without CPPO *n* = 28	*p*-value*
1	20 (74.1%)	17 (60.7%)	.391
2	4 (14.8%)	9 (32.1%)	.205
3	2 (7.4%)	1 (3.6%)	.611
4	0 (0.0%)	1 (3.6%)	1.000
5	0 (0.0%)	0 (0.0%)	n/a
6	1 (3.7%)	0 (0.0%)	.491

Notes.

* *p*-values are calculated using Fisher’s Exact Test.

**Table 5.** Summary of operative-related outcomes.

Characteristic	Intervention group (*n* = 27)	Control group (*n* = 28)	*p*-value
Intubation time (s)*	26 (12 − 657)	55.5 (7 − 467)	.106
Number of intubation attempts*	1 (1 − 6)	1 (1 − 4)	.403
MCLS (1-2a:2b-4)**	16:11	14:14	.491
Tissue Damage (Yes:No)***	0:27	6:22	.023

Note.

* *p*-value is calculated using Mann-Whitney *U* test.

^**^ Values are expressed as median (min-max). *p*-value is calculated using Pearson chi-square test.

^***^ Values are expressed as median (min-max). *p*-value is calculated using Fisher’s exact test.

No tissue damage was observed in the CPPO group, while there were 21.4% of patients (*n* = 6) with tissue damage in the control group (Table 2). Based on data summarized in Table 2, the use of the 3D protective CPPO was found to be correlated with less tissue damage during airway management (*χ*^2^ = 6.494, df = 1, *p* = .011; FET: *p* = .023). Subsequently, two separate assessments were performed to evaluate correlations between the use of the CPPO and the tissue damage incidence for the patients with the less and more severe cleft type. Therefore, patients from both intervention and control groups were categorized based on type of cleft into two sub-groups (1. Less severe clefts: U/BCLA; 2. More severe clefts: U/BCLAP) to comprehensively evaluate the impact of this factor on tissue damage. Corresponding Pearson chi-square and Fisher’s exact tests found no significant effect of the CPPO usage on the tissue damage incidence in the instances associated with less severe clefts (*χ*^2^ = 2.012, df = 1, *p* = .156; FET: *p* = .474). On the contrary, some effect was suggested in the instances of more severe clefts; however, this result was not conclusive due to the small number of samples (*χ*^2^ = 4.500, df = 1, *p* = .034; FET: *p* = .104).

The evaluation based on the MCLS in both monitored groups of patients is shown in Table 3. In patients with CPPO, difficult intubation was present in only 40.7%, in contrast to the control group without CPPO, in which difficult intubation occurred in 50%. The difference was not statistically significant (*χ*^2^ = 2.449, df = 1, *p* = .118; FET: *p* = .239). The median intubation time in the intervention group was 26.0 s (min-max: 12–657 sec.) with a median number of intubation attempts being 1.0 (min-max: 1–6). The median intubation time in the control group was 55.5 s (min-max: 7–467 sec.) with a median number of intubation attempts being 1.0 (min-max: 1–4). The numbers of intubation attempts are summarized in more detail in Table 4. The comparison of the intubation times yielded no statistically significant difference between both intervention and control groups (*n*_1_=27, *R*_1_=663, *n* = 28, *R*_2_=877, *U* = 285, *Z* = −1.557, *p* = .119). Similarly, the numbers of intubation attempts did not differ significantly in both groups (*χ*^2^ = 4.483, df = 4, *p* = .344).

## Discussion

To the best of our knowledge this is the first study describing the usage of individualized protective CPPO specifically in newborns with CLA/P in general anesthesia during intubation for cleft lip repair surgery.

The centralization of care for children with cleft lip and palate is essential to ensure access to specialized resources and expertise, which significantly improves treatment outcomes. An important aspect of this approach is the involvement of a multidisciplinary team, including surgeons, orthodontists, speech therapists, and psychologists, to effectively address the complex needs of patients [2]. An anesthesiologist experienced in the field of clefts is an essential part of a cleft center, ensuring safe and effective management of patients during surgical procedures.

Anesthesia for cleft lip and palate surgery carries a high risk of complications, especially difficult airway management [15–17]. The increased incidence of anesthetic complications in children with cleft lip and palate can be attributed to many factors. These include higher airway resistance, higher incidence of respiratory infections, nutritional deficiencies, developmental anomalies, and anatomical features such as micrognathia, macroglossia, and jaw hypoplasia [18].

Anatomical defects increase the risk of difficult laryngoscopy and intubation [19], which is confirmed by some studies. Gunawardana reported that difficult laryngoscopy was significantly associated with age under 6 months, bilateral cleft lip, and retrognathia [20]. Similar results were reported by Xue et al. who reported difficult laryngoscopy in infants with cleft lip and palate, where age <6 months, combined bilateral cleft lip and palate, and micrognathia were significant risk factors. They reported difficult intubation primarily in infants with laryngoscopic findings of MCLS 3 and 4 [5,21,22].

Patient′s age at the time of cleft lip surgery and cleft type itself may require a specific anesthesiologic management [21]. Especially intubation is often difficult due to the defect′s nature with high incidence of complications such as difficult airway management, desaturation, laryngospasm, or bradycardia [6]. According to experience of our Cleft Center, tissue damage (e.g. soft tissue of the lip, alveolar arch, palate, nasal septum, skeleton of the premaxilla and nasal septum) occurs during intubation in some of patients and is mainly caused by the laryngoscopic blade during intubation [11].

Previous studies have explored the use of artificial devices to cover defects in the alveolus and palate [23]. Karayazgan et al. [7] proposed the use of a palatal lift obturator made of a flexible and durable material. The overlapping flexible tulle attached to the palatal plate was created from an impression of the defect covering the soft tissue [5]. Another randomized study by Mahmoud et al. [6] focused on facilitating intubation, was conducted with children aged 9 months to 6 years. A hard gum shield, approximately shaped to fit the palate, was used during laryngoscopy and intubation, with positive observations improving many monitored factors, such as intubation time [6,7]. The pilot study of usage of an individualized palate protective tray using 3D printing and its influence on tissue protection and intubation procedure in cleft patients in general was described by Richtrova in 2023 [11]. The uniqueness of this study is based on the 3D-printed palatal obturator for intubation in newborns during cleft lip surgery, which has not been described in such a unique group of patients in the literature yet.

Our study demonstrates that using CPPO during intubation in newborns significantly reduces the risk of oral tissue injury. A significant difference was observed particularly in cases of more severe clefts, suggesting that CPPO may be especially beneficial in these patients. However, this observation cannot be considered conclusive due to the small number of samples. There was no significant difference between the two groups regarding intubation time and the number of intubation attempts. Although not statistically significant a trend toward fewer intubation attempts and a lower incidence of difficult intubation (MCLS 2b, 3, and 4) was observed in the CPPO group. This observation is relevant as it suggests the CPPO does not add complexity or delay to the intubation procedure. Moreover, anesthesiologists provided subjective feedback indicating improved ease of manipulation of the laryngoscopic blade when using CPPO.

According to the available literature, anesthetic management does not differ for left-sided and right-sided facial clefts. It always depends on the anatomical specifics of the cleft and its size and extent. In principle, the anesthetic procedure will be adapted to the individual needs of the patient and the specifics of the surgical procedure rather than whether the cleft is on the right or left side. However, it would be appropriate to determine these specifics in future studies.

Anesthetic management differs between cleft lip, cleft palate, and combined cleft lip and palate due to variations in airway anatomy and surgical complexity. Cleft lip and palate repair can lead to significant difficulties in mask ventilation and intubation due to airway leaks and structural abnormalities. Combined cleft lip and palate cases require a more tailored approach, as they inherit airway challenges from both conditions, often necessitating advanced intubation techniques [5,9,24]. Understanding these differences is crucial for optimizing perioperative management and reducing anesthesia-related complications in cleft patients.

### Limitations

The Cleft Center of the University Hospital has established a specialized team of anesthesiologists who focus on treating children with clefts, resulting in a very high level of experience. However, to generalize the study’s conclusions, it would be beneficial to apply CPPO in centers where cleft lip, particularly in newborns, is not as common a diagnosis. Importantly, this is a single-center study conducted in a highly specialized setting, which may limit the external validity and generalizability of the results.

One of the main limitations of our study is the relatively small sample size (*n* = 55), which may have impacted the statistical power of our analyses. While we observed a significant reduction in tissue damage associated with the use of CPPO (*p* = .023), the lack of statistical significance in other comparisons, such as intubation difficulty and intubation time, could be attributed to the limited number of subjects. The limited cohort size primarily reflects the unique and relatively rare nature of the neonatal patient population under investigation. A larger sample size might allow for more precise estimates and potentially reveal significant differences that were not detectable in our current dataset. Future studies with expanded subject groups are warranted to validate our findings and further explore the potential benefits of CPPO in clinical practice.

## Conclusion

The use of CPPO in newborns with cleft lip, alveolus, and palate reduces tissue damage at the cleft site during intubation. Our data showed no statistically significant difference in airway visualization, the number of intubation attempts, or intubation duration. This demonstrates that the CPPO provides its key benefit of tissue protection without compromising the safety or efficiency of the intubation process. CPPO may be considered a valuable adjunct during intubation for patients with severe cleft lip and palate, particularly when the cleft affects both the lip and palate or is notably wide.

## Supplementary Material

Supplemental Material

CONSORT_revised.doc

Appendix_clear.docx

## Data Availability

The data and materials supporting the results or analyses presented in this paper are available upon reasonable request by contacting the corresponding author.
