# Supplementary figures and images for: Customized protective palatal obturator for intubation in newborns in cleft lip surgery: a randomized controlled trial
Source: Ann Med. 2025 Sep 22;57(1):2561802. doi: 10.1080/07853890.2025.2561802 (PMC12456041; doi:10.1080/07853890.2025.2561802)

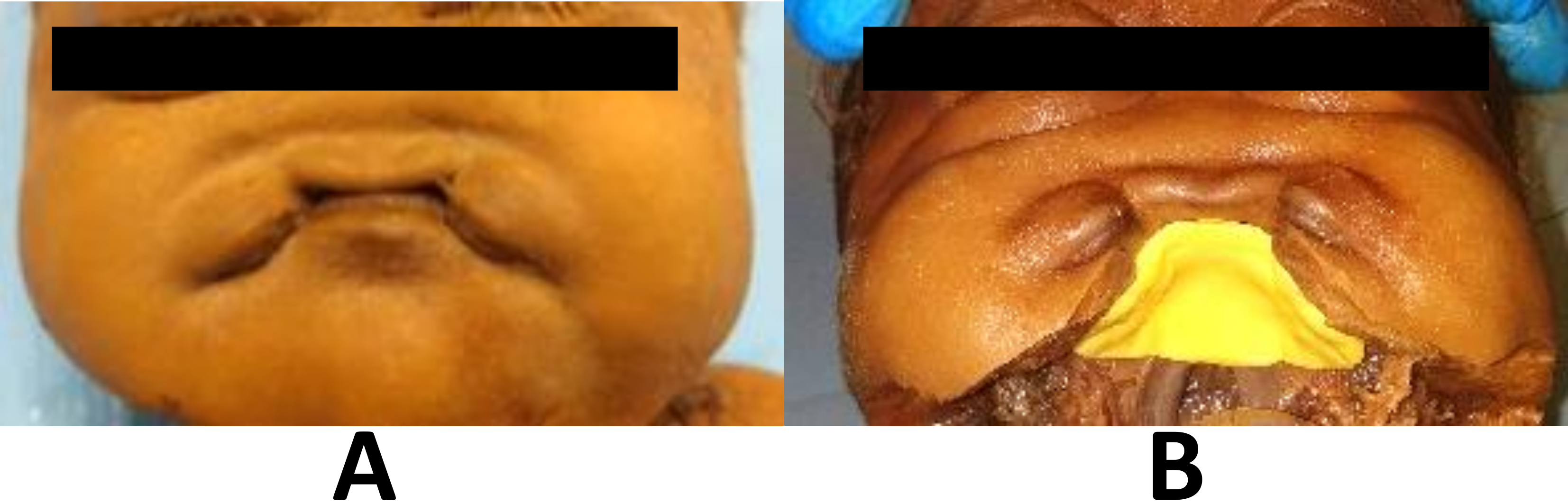

Supplement: Supplemental Material [file IANN_A_2561802_SM1684.jpg]
