# Supplementary material for: Customized protective palatal obturator for intubation in newborns in cleft lip surgery: a randomized controlled trial
Source: Ann Med. 2025 Sep 22;57(1):2561802. doi: 10.1080/07853890.2025.2561802 (PMC12456041; doi:10.1080/07853890.2025.2561802)
Supplement: Appendix_clear.docx [file IANN_A_2561802_SM2873.docx]

**APPENDIX**

Pilot study

This appendix details the pilot study conducted to validate the design and fabrication workflow for the CPPO before its implementation in a clinical trial. This preliminary work was performed on a cadaveric specimen with a cleft lip and palate to assess feasibility of the proposed project (the pilot study was approved by Ethics Committee of Faculty of Medicine, Masaryk university, Czech Republic; approval number MU-IS/163699/2024/2411682/LF).

The cadaveric specimen used in the pilot study was a male neonate with an atypical cleft lip and palate and a hypoplastic premaxilla (Figure A1 (A)), dated to the second half of the 20th century. It had been fixed in a 40% aqueous solution of formaldehyde (CH2O). First, a 3D scan of the upper jaw including cleft palate and alveolar process was performed using an intraoral 3D scanner (3SHAPE TRIOS 3). A virtual design of the protective obturator was then created using CAD software (MAGICS and Meshmixer). To facilitate access and test the CPPO fit, dissections were made from the oral commissures toward the lower edge of the auricles. The CPPO fitted precisely on the upper jaw giving a stable surface for tissue protection during intubation (Figure A1 (B)). The entire process, from scanning to the final protective obturator preparation, took 24 hours.

**Legends:**

Figure A1. Cadaveric pilot study: (A) The cadaveric specimen with an atypical cleft lip and palate. (B) The custom-fitted CPPO placed on the upper jaw.
